# Diastolic Blood Pressure is a Risk Factor for Peri-procedural Stroke Following Carotid Endarterectomy in Asymptomatic Patients

**DOI:** 10.1016/j.ejvs.2017.02.004

**Published:** 2017-05

**Authors:** D.D. de Waard, G.J. de Borst, R. Bulbulia, A. Huibers, A. Halliday, the Asymptomatic Carotid Surgery Trial-1 Collaborative Group

**Affiliations:** a Nuffield Department of Surgical Sciences, University of Oxford, Level 6 John Radcliffe Hospital, Oxford OX3 9DU, UK; b Department of Vascular Surgery, University Medical Centre Utrecht, PO Box 85500, Utrecht, The Netherlands; c Medical Research Council Population Health Research Unit, Clinical Trial Service Unit & Epidemiological Studies Unit, Nuffield Department of Population Health, Richard Doll Building, Old Road Campus Roosevelt Drive, Oxford OX3 7LF, UK

**Keywords:** Blood pressure, Carotid artery stenosis, Carotid endarterectomy, Periprocedural stroke

## Abstract

**Objective/Background:**

Carotid endarterectomy (CEA) prevents future stroke, but this benefit depends on detection and control of high peri-operative risk factors. In symptomatic patients, diastolic hypertension has been causally related to procedural stroke following CEA. The aim was to identify risk factors causing peri-procedural stroke in asymptomatic patients and to relate these to timing of surgery and mechanism of stroke.

**Methods:**

In the first Asymptomatic Carotid Surgery Trial (ACST-1), 3,120 patients with severe asymptomatic carotid stenosis were randomly assigned to CEA plus medical therapy or to medical therapy alone. In 1,425 patients having their allocated surgery, baseline patient characteristics were analysed to identify factors associated with peri-procedural (< 30 days) stroke or death. Multivariate analysis was performed on risk factors with a *p* value < .3 from univariate analysis. Event timing and mechanism of stroke were analysed using chi-square tests.

**Results:**

A total of 36 strokes (27 ischaemic, four haemorrhagic, five unknown type) and six other deaths occurred during the peri-procedural period, resulting in a stroke/death rate of 2.9% (42/1,425). Diastolic blood pressure at randomisation was the only significant risk factor in univariate analysis (odds ratio [OR] 1.34 per 10 mmHg, 95% confidence interval [CI] 1.04–1.72; *p* = .02) and this remained so in multivariate analysis when corrected for sex, age, lipid lowering therapy, and prior infarcts or symptoms (OR 1.34, 95% CI 1.05–1.72; *p* = .02). In patients with diastolic hypertension (> 90 mmHg) most strokes occurred during the procedure (67% vs. 20%; *p* = .02).

**Conclusion:**

In ACST-1, diastolic blood pressure was the only independent risk factor associated with peri-procedural stroke or death. While the underlying mechanisms of the association between lower diastolic blood pressure and peri-procedural risk remain unclear, good pre-operative control of blood pressure may improve procedural outcome of carotid surgery in asymptomatic patients.

**What this paper adds**

The net benefit of carotid revascularisation is highly dependent on procedural risk. In this paper, diastolic blood pressure is shown to be an independent risk factor for peri-procedural stroke/death following carotid endarterectomy (CEA) in the Asymptomatic Carotid Surgery Trial-1. This is in agreement with a recent study in symptomatic patients undergoing CEA. Better blood pressure regulation in the peri-procedural period has the potential to make carotid surgery safer, especially for asymptomatic patients where there is time to treat hypertension pre-operatively.

## Introduction

Large randomised trials in symptomatic and asymptomatic patients have shown that carotid endarterectomy (CEA) prevents stroke.^1^, ^2^ The overall benefit depends on interventional hazard and the long-term benefit from stroke risk reduction. To reduce interventional hazard, it is important to understand mechanisms causing peri-procedural stroke and treat factors associated with increased risk.^3^ For asymptomatic patients there is usually time for optimisation before intervention, thereby minimising the hazards of surgery.

Haemodynamic instability has been identified previously as a risk factor for surgery, and remains important, even though blood pressure control has improved.^4^, ^5^, ^6^ Recently, the peri-procedural hazards of poor diastolic blood pressure (DBP) control were highlighted by a substudy from the symptomatic International Carotid Stenting Study, which found DBP to be the single independent risk factor for peri-procedural complications (relative risk 1.30 for each +10 mmHg DBP, 95% confidence interval [CI] 1.02–1.66; *p* = .04) in symptomatic patients.^7^

In this study, using data from the large Asymptomatic Carotid Surgery Trial-1 (ACST-1), the aim was to: (i) identify modifiable risk factors for peri-procedural stroke or death following CEA; and (ii) relate these risk factors to the timing and mechanism of stroke.

## Methods

### Patient selection

The ACST-1 protocol has been described previously.^8^ The trial included 3,120 patients with unilateral or bilateral carotid stenosis, who were randomised to either immediate CEA with medical therapy or to medical therapy alone. Only patients without ipsilateral neurological symptoms in the past 6 months were entered, and patients randomised to immediate surgery were expected to have their operation as soon as reasonably possible. Choice of surgical and anaesthetic technique was left to the surgeons' discretion. All surgeons' track records for CEA had been approved by the trial Technical Management Committee.

Only patients randomised to immediate intervention and who underwent ipsilateral CEA were included in this post-hoc study.

### Outcome events

Patients were assessed by a neurologist following surgery and before discharge. Strokes and deaths within 30 days of CEA were considered to have been caused by or related to the procedure. Two independent members of the endpoint adjudication committee reviewed all events reported to the trial office. Any disagreement was resolved through discussion. Strokes were classified according to type (ischaemic or haemorrhagic), severity (non-disabling, disabling, fatal), laterality (ipsilateral, contralateral, vertebrobasilar), and timing (intra-procedural, day 0 post-procedural, day 1–30).

### Classification of stroke mechanism

In a previous study involving patients from ACST-1, the most likely pathophysiological mechanisms for peri-procedural stroke were determined.^9^ Ischaemic strokes were classified as follows: (i) carotid-embolic, if there was direct visualisation of an intracranial embolus reported on angiographic brain imaging, or when there was a clear association between onset of symptoms and shunt insertion; (ii) haemodynamic, if there was intra- or post-procedural bradycardia (< 40 beats per minute), asystole, or any hypotension requiring treatment; (iii) thrombosis or occlusion of the carotid artery if a residual stenosis or occlusion of the internal carotid artery (ICA) was found by either imaging or re-exploration, irrespective of the occurrence of an embolic or haemodynamic event; (iv) hyperperfusion, when seizures, a throbbing headache, or neurological deficit occurred in conjunction with intracerebral haemorrhage or cerebral oedema on post-procedural brain imaging; (v) cardio-embolic, if atrial fibrillation was detected on electrocardiography immediately after stroke; (vi) undetermined: (a) probably carotid embolic or haemodynamic, in case the ICA was found to be patent, but there was no radiographic evidence to classify stroke carotid-embolic or haemodynamic; (b) probably carotid-embolic or thrombotic occlusion, when stroke occurred intra-procedurally in the absence of any haemodynamic or cardio-embolic event; (c) all other strokes of undetermined origin.

### Statistical analysis

Baseline patient characteristics were collected at time of randomisation and analysed as potential risk factors for the combined outcome of peri-procedural stroke or death. Blood pressure was measured according to local hospital protocol (usually bilateral) and the highest value for systolic blood pressure (SBP) and DBP was used for risk factor analysis. Age, blood pressure, and cholesterol were analysed as continuous variables, while ipsi- and contralateral degree of stenosis were analysed as categorical variables. Patients with missing data were excluded from analysis. Univariate binomial logistic regression analysis was used to calculate odds ratios (OR) with 95% Wald CIs for occurrence of peri-procedural stroke or death. A multivariate logistic regression analysis was performed using variables with a *p* value <.30 in univariate analysis. Chi-square analysis of timing of stroke was performed comparing normotensive and hypertensive patients. Hypertension was defined as a DBP of > 90 mmHg and a SBP > 140 mmHg, according to the National Institute for Health and Care Excellence guidelines.^10^ Factors with *p* values < .05 were considered significant for all analyses.

## Results

### Study population

In 1,560 patients allocated to immediate surgery, CEA was eventually undertaken in 1,425/1,560 (91.3%). Most patients (893/1,425; 62.7%) were operated on within 6 weeks of randomisation, and almost all who eventually had their allocated surgery did so within 1 year (1,388/1,425; 97.4%). Two thirds of participants were men (66.3%) and the mean ± SD age of all patients was 69 ± 7.5 years. Mean ± SD stenosis in the operated carotid artery was 80 ± 11%. The mean ± SD SBP and DBP at randomisation were 154 ± 22 mmHg and 83 ± 11 mmHg, respectively. Baseline patient characteristics and their peri-procedural risk of stroke or death are shown in Table 1.

### Outcome events

Peri-procedural stroke or death was uncommon in ACST-1 (42/1,425; 2.9%) and events are shown in Table 2. Most events were strokes (36/42; 85.7%) and these were usually ischaemic (27/36; 75.0%) and ipsilateral to the treated carotid artery (28/36; 77.8%). Four strokes were haemorrhagic and five could not be classified. Over half were fatal (11/36; 30.6%) or disabling (9/36; 25.0%). Most non-stroke deaths were due to cardiac causes (5/6; 83.3%).

### Risk factors for peri-procedural stroke or death

**Figure 1:**
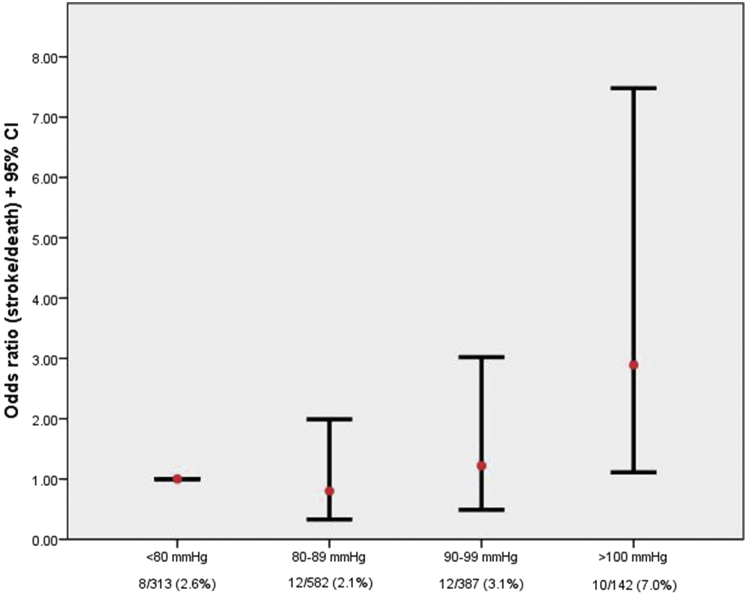
Odds ratios and observed risk for peri-procedural stroke/death (95% confidence interval [CI]) for increasing levels of diastolic blood pressure measured at baseline.

Results from univariate analysis of baseline characteristics are summarised in Table 1. DBP was the only risk factor significantly associated with stroke or death within 30 days (OR 1.34 per 10 mmHg, 95% CI 1.04–1.72; *p* = .02). The risk of peri-procedural stroke or death was almost twice as high in patients with diastolic hypertension (≥ 90 mmHg) than in patients with DBP < 90 mmHg (4.2% vs. 2.2%) (Fig. 1). None of the other risk factors, including age (OR 1.14 per 5 years, 95% CI 0.92–1.41; *p* = .24), female sex (OR 1.65, 95% CI 0.89–3.07; *p* = .11), and ipsilateral stenosis ≥ 70% (OR 4.33, 95% CI 0.59–31.71; *p* = .15), were significantly associated with peri-procedural stroke or death in univariate analysis.

After adjustment for age, sex, lipid lowering therapy, ipsilateral degree of stenosis, and prior symptoms and/or infarcts (Table 3), DBP remained the only independent risk factor for peri-procedural stroke or death (OR 1.34 per 10 mmHg, 95% CI 1.05–1.72; *p* = .02).

### Timing of stroke and stroke mechanism

The mechanism likely to have caused stroke is summarised in Table 4. For five patients the type of stroke (ischaemic or haemorrhagic) could not be determined and were therefore excluded from Table 4. A definite stroke mechanism could be determined for most events (21/31; 67.7%) and for four others a likely mechanism was identified.

The median time from procedure to onset of neurological symptoms was 0 days for ischaemic stroke (range 0–26 days), with 11/27 (40.7%) occurring during the procedure. Haemorrhagic stroke occurred after the day of surgery in all cases (median 4.5 days, range 1–8 days). In patients with diastolic hypertension (DBP ≥ 90 mmHg), stroke occurred more frequently during surgery than in normotensive (DBP < 90 mmHg) patients (67% vs. 20%; *p* = .02).

## Discussion

It was found that baseline diastolic blood pressure (i.e., prior to CEA) was the only identifiable independent risk factor for peri-procedural stroke or death in this group of asymptomatic patients.

Hypertension is an important cause of stroke, with risk rising continuously as blood pressure increases above 115/75 mmHg.^11^, ^12^ Evidence from clinical trials shows that this risk is reversible with control of blood pressure.^13^, ^14^

SBP is important for autoregulation of cerebral blood flow.^15^ Arterial wall stiffening, part of the natural ageing process, is sometimes associated with isolated systolic hypertension (ISH). In a meta-analysis, patients with ISH also had an increased risk of stroke (1.22, 95% CI 1.04–1.40; *p* = .02).^16^

High pulse pressure (PP) is associated with the presence of carotid artery stenosis, but is more strongly related to subsequent cardiac events than to stroke.^17^ However, mean arterial pressure (MAP) is a better predictor of cerebrovascular events,^18^, ^19^ and MAP is more dependent on DBP (MAP = 1/3 SBP + 2/3 DBP), suggesting that DBP may have an important role in stroke. This is consistent with the results, where DBP was the only clear predictor associated with peri-procedural stroke or death, while there was a much weaker association with MAP and no association with SBP or PP (Table 1).

Although the association between hypertension and long-term stroke risk is well known, the effects of hypertension during the peri-procedural period associated with carotid intervention are less clear. Post-procedural hypertension is common during and after CEA, and often needs treatment. Baroreceptor sensitivity is decreased in patients with carotid atheroma and may be further compromised by surgical trauma to sensory nerves within the arterial wall.^6^, ^20^ Haemodynamic instability during the procedure and in the early post-operative period has been reported in just over half (54%) of patients undergoing CEA,^21^ and is more likely to occur when CEA is performed under general anaesthesia.^22^ Sudden blood pressure changes can cause ischaemic (and haemorrhagic) stroke, especially when autoregulation is diminished and arterial stiffness is high.^23^

Haemodynamic disturbances have been shown to be an important mechanism of peri-procedural stroke during CEA in symptomatic patients.^4^ In the present study, almost half of strokes (10/21) had similar causes. However, there were a number of strokes of uncertain aetiology in the hypertensive group (seven of 12), which prevented any more definite conclusion being drawn from the comparison of stroke mechanism between hypertensive and normotensive patients.

Although more strokes occurred during CEA in patients with diastolic hypertension than in normotensive patients, the mechanism for half of intra-procedural strokes in the hypertensive group remained unclear. Severe hypotension caused by manipulation of the carotid sinus and by baroreceptor dysfunction and individual factors may make patients vulnerable to intra-procedural hypotension.^20^, ^23^, ^24^

Post-operative hypertension, often associated with pre-operative hypertension, may be preventable and caused three of four haemorrhagic strokes.^6^, ^25^ Monitoring of cerebral blood flow with transcranial Doppler measurements during and after CEA has been shown to be helpful in the early identification and treatment of hyperperfusion syndrome (> 100% increase in cerebral blood flow).^26^, ^27^

The present results suggest that more attention should be paid to treating hypertension before surgery. Current international guidelines recommend blood pressure is kept < 140/90 mmHg in the general population and < 130/80 mmHg in patients with diabetes or patients with renal impairment.^10^ These guidelines may change following results from the SPRINT trial, showing that when all patients were treated to a target SBP of < 120 mmHg, risks for all cardiovascular events fell; however, patients with borderline pressure dependent organ perfusion may be at increased risk of serious events, a caveat that may be particularly relevant for patients with tight carotid stenosis.^14^

In the present study, 65% of patients were on antihypertensive treatment, but often the single measurement given at randomisation exceeded guideline targets. Mean DBP and SBP were significantly higher in treated patients than patients without antihypertensive treatment (DBP: 84 vs. 81 mmHg [*p* < .01]; SBP: 156 vs. 149 mmHg [*p* < .01]). Untreated diastolic and systolic hypertension was present in 10% and 19% of patients, respectively. Even in a clinical trial, albeit one with “real life” practice, blood pressure control before surgery can be improved.

As diastolic hypertension was only recorded in 22/42 (52.4%) of patients that had an event, other factors could also be important. In asymptomatic patients there is no necessity for immediate revascularisation, and care should be taken to prevent peri-procedural stroke. Guidelines on carotid revascularisation currently do not provide a pre-operative target BP.^28^ Future studies should focus on trying to define optimal DBP targets and on the potential benefits of pre-operative lowering of DBP, even though these may be dependent on individual patient cerebral perfusion.

This study has several limitations. Firstly, blood pressure was only measured once at randomisation. With a single measurement, associations will be underestimated owing to regression dilution bias, and repeated measurements should improve accuracy. Secondly, peri-procedural stroke was uncommon in ACST-1. Therefore, other contributors to peri-procedural stroke or death may not have been detected. However, the findings are consistent with those of another trial of CEA in symptomatic patients, making it less likely that these results are a chance finding. For a more robust analysis of risk factors, studies of large registries with hundreds of thousands of patients with more peri-procedural events are needed.

## Conclusion

In ACST-1, DBP was an independent risk factor for peri-procedural stroke or death in asymptomatic patients. Risk of intra-procedural stroke is higher in hypertensive patients. While the underlying mechanisms of the association between DBP and peri-procedural risk remains unclear, good pre-operative control of blood pressure may improve peri-procedural outcome of carotid surgery in asymptomatic patients.

## Figures and Tables

**Table 1:** Baseline characteristics and univariate logistic regression.

	No. events/no. patients (%)	Unadjusted OR (95% CI)	*p*
Age (per 5 y)			
	42/1,425 (2.9)	1.14 (0.92–1.40)	.24
Sex			
Male	23/945 (2.4)	1.00	.11
Female	19/480 (4.0)	1.65 (0.89–3.07)	
Diabetes			
No	32/1,136 (2.8)	1.00	.56
Yes	10/289 (3.5)	1.24 (0.60–2.55)	
CHD			
No	26/947 (2.7)	1.00	.53
Yes	16/478 (3.3)	1.23 (0.65–2.31)	
Antihypertensive treatment			
No	14/498 (2.8)	1.00	.82
Yes	28/927 (3.0)	1.08 (0.56–2.07)	
Antiplatelet treatment			
No	4/146 (2.7)	1.00	.88
Yes	38/1,279 (3.0)	1.09 (0.38–3.09)	
Lipid lowering therapy			
No	25/967 (2.6)	1.00	.24
Yes	17/458 (3.7)	1.45 (0.78–2.72)	
Baseline cholesterol (per 1 mmol/L)			
	40/1,355 (3.0)	0.97 (0.76–1.25)	.84
SBP (per 10 mmHg)			
	42/1,424 (2.9)	0.99 (0.86–1.14)	.93
DBP (per 10 mmHg)			
	42/1,424 (2.9)	1.34 (1.04–1.72)	.02
Pulse pressure (per 10 mmHg)			
	42/1,424 (2.9)	0.88 (0.74–1.05)	.15
MAP (per 10 mmHg)			
	42/1,424 (2.9)	1.16 (0.92–1.45)	.21
Stenosis in treated artery (%)			
< 70	1/133 (0.8)	1.00	.15
≥ 70	41/1,292 (3.2)	4.33 (0.59–31.71)	
Contralateral stenosis (%)			
0–49	22/851 (2.6)	1.00	.79
50–69	10/280 (3.6)	1.40 (0.65–2.98)	
70–99	5/159 (3.1)	1.22 (0.46–3.28)	
Occlusion	5/135 (3.7)	1.45 (0.54–3.89)	
Echolucency			
Not echolucent	11/335 (3.3)	1.00	.79
Echolucent	13/356 (3.7)	1.12 (0.49–2.53)	
Infarction on imaging (CT or MRI)			
No infarction	19/619 (3.1)	1.00	.38
Infarction	12/285 (4.2)	1.39 (0.66–2.90)	
Prior symptoms any side and/or infarction on imaging (CT/MRI)			
No	17/755 (2.3)	1.00	.10
Yes	25/670 (3.7)	1.68 (0.90–3.14)	

*Note.* OR = odds ratio; CI = confidence interval; CHD = coronary heart disease; SBP = systolic blood pressure; DBP = diastolic blood pressure; MAP = mean arterial pressure; CT = computed tomography; MRI = magnetic resonance imaging.

**Table 2:** Peri-procedural stroke and death in ACST-1.

Outcome events	*n*
Stroke Type	
Ischaemic	27
Haemorrhagic	4
Unknown	5
Stroke Laterality	
Ipsilateral	28
Contralateral	7
Vertebrobasilar	1
Stroke Severity	
Fatal	10
Disabling	8
Non-disabling	18
Cardiac death	5
Other death	1
Total	42

**Table 3:** Multivariate logistic regression of peri-procedural stroke or death with predictors *p* < .3 in univariate analysis.

	Adjusted OR (95% CI)	*p*
Age (per 5 y)		
	1.14 (0.92–1.42)	.22
Sex		
Male	1.00	.18
Female	1.54 (0.82–2.87)	
Lipid lowering therapy		
No	1.00	.21
Yes	1.51 (0.80–2.86)	
DBP (per 10 mmHg)		
	1.34 (1.05–1.72)	.02
Stenosis in treated artery (%)		
< 70	1.00	.14
≥ 70	4.56 (0.62–33.85)	
Prior symptoms and/or infarction on imaging		
No	1.00	.08
Yes	1.77 (0.94–3.31)	

*Note.* OR = odds ratio; CI = confidence interval; DBP = diastolic blood pressure.

**Table 4:** Stroke mechanism according to procedural time interval.

Ischaemic stroke	Total		Day 0, intra-procedural		Day 0, after procedure		Days 1–30	
	< 90 mmHg	> 90 mmHg	< 90 mmHg	> 90 mmHg	< 90 mmHg	> 90 mmHg	< 90 mmHg	> 90 mmHg
	*n* = 15	*n* = 12	*n* = 3	*n* = 8	*n* = 6	*n* = 3	*n* = 6	*n* = 1
1. Carotid-embolic	2 (13)	2 (17)	1 (33)	1 (13)	1 (17)	1 (33)	0 (0)	0 (0)
2. Haemodynamic	3 (20)	1 (8)	1 (33)	1 (13)	1 (17)	0 (0)	1 (17)	0 (0)
3. Thrombosis/occlusion of artery	6 (40)	1 (8)	1 (33)	0 (0)	4 (67)	0 (0)	1 (17)	1 (100)
4. Hyperperfusion	0 (0)	0 (0)	0 (0)	0 (0)	0 (0)	0 (0)	0 (0)	0 (0)
5. Cardio-embolic	2 (13)	1 (8)	0 (0)	1 (13)	0 (0)	0 (0)	2 (33)	0 (0)
Most likely mechanism 1 or 2	0 (0)	3 (25)	0 (0)	3 (38)	0 (0)	0 (0)	0 (0)	0 (0)
Most likely mechanism 1 or 3	0 (0)	1 (8)	0 (0)	1 (13)	0 (0)	0 (0)	0 (0)	0 (0)
Undetermined	2 (13)	3 (25)	0 (0)	1 (13)	0 (0)	2 (67)	2 (33)	0 (0)

Haemorrhagic	Total		Day 0, intra-procedural		Day 0, after procedure		Days 1–30	
	< 90 mmHg	> 90 mmHg	< 90 mmHg	> 90 mmHg	< 90 mmHg	> 90 mmHg	< 90 mmHg	> 90 mmHg
	*n* = 2	*n* = 2	*n* = 0	*n* = 0	*n* = 0	*n* = 0	*n* = 2	*n* = 2
Hyperperfusion	2 (100)	1 (50)	0 (0)	0 (0)	0 (0)	0 (0)	2 (100)	1 (50)
Undetermined	0 (0)	1 (50)	0 (0)	0 (0)	0 (0)	0 (0)	0 (0)	1 (50)

*Note.* Results shown by diastolic blood pressure groups. In five patients type of stroke could not be determined and they were therefore excluded from this table. Data are *n* (%).
